# A Critical Analysis of Rejection in Vascularized Composite Allotransplantation: Clinical, Cellular and Molecular Aspects, Current Challenges, and Novel Concepts

**DOI:** 10.3389/fimmu.2013.00406

**Published:** 2013-11-25

**Authors:** Karim A. Sarhane, Sami H. Tuffaha, Justin M. Broyles, Amir E. Ibrahim, Saami Khalifian, Pablo Baltodano, Gabriel F. Santiago, Mohammed Alrakan, Zuhaib Ibrahim

**Affiliations:** ^1^Department of Plastic and Reconstructive Surgery, Johns Hopkins University School of Medicine, Baltimore, MD, USA; ^2^Department of Plastic and Reconstructive Surgery, M.D. Anderson Cancer Center, Houston, TX, USA; ^3^Department of Otolaryngology, Head and Neck Surgery, Walter Reed National Military Medical Center, Bethesda, MD, USA

**Keywords:** vascularized composite allotransplantation, transplant rejection, Banff classification, reconstructive transplantation, hand/face transplantation

## Abstract

Advances in microsurgical techniques and immunomodulatory protocols have contributed to the expansion of vascularized composite allotransplantation (VCA) with very encouraging immunological, functional, and cosmetic results. Rejection remains however a major hurdle that portends serious threats to recipients. Rejection features in VCA have been described in a number of studies, and an international consensus on the classification of rejection was established. Unfortunately, current available diagnostic methods carry many shortcomings that, in certain cases, pose a great diagnostic challenge to physicians especially in borderline rejection cases. In this review, we revisit the features of acute skin rejection in hand and face transplantation at the clinical, cellular, and molecular levels. The multiple challenges in diagnosing rejection and in defining chronic and antibody-mediated rejection in VCA are then presented, and we finish by analyzing current research directions and novel concepts aiming at improving available diagnostic measures.

## Introduction

Reconstructive transplantation using vascularized composite tissue was introduced to clinical reality in 1998 with the first hand transplant (1), and in 2005 with the first face transplant (2). These were built on many years of basic and preclinical research aiming largely at establishing the immunobiology of vascularized composite tissue allotransplantation (VCA). This type of transplantation is unique as it carries complex immunologic challenges. Effectively, VCA consists of various heterogeneous tissue types of different antigenicity, including skin, vasculature, muscle, cartilage, tendon, nerve, bone, bone marrow (BM), and vascularized BM. The high immunogenicity of the skin and, to a lesser extent vasculature, necessitate the utilization of multi-immunosuppressive drug regimens (sometimes administered in high doses) in order to prevent skin rejection and graft failure. Initial experiences with hand transplantation in 1964, although technically successful, failed to overcome such immunologic challenges; severe rejection occurred 2 weeks postoperatively, and required re-amputation (3). Subsequently investigators were urged to withhold further clinical trials until more basic science research is conducted.

Today, up to 26 facial and close to 100 hand allotransplantations have been performed worldwide with excellent short-to-intermediate functional and immunological outcomes. Rejection however remains a major obstacle to broader application of VCA, and poses serious threats to recipients. About 85% of all patients experienced at least one episode of acute skin rejection within the first postoperative year, and as much as 56% experienced multiple episodes (4). Rejection features in VCA have been studied at the clinical, cellular, and molecular levels, and an international standardized classification system for the diagnosis and grading of skin rejection was established (5). However, this system carries multiple inherent weaknesses due its almost exclusive reliance on non-specific clinicopathologic cues. Furthermore, the cellular and molecular basis of skin rejection in VCA, although partially delineated, remain largely unknown. Hence the diagnosis of rejection in VCA is a major challenge. The aim of this review is to present the scope of this challenge, highlighting the current unmet diagnostic needs in VCA and analyzing current and future research directions working towards overcoming such hurdles. We will first revisit the salient features of acute skin rejection in hand and face transplantation, underlining their close similarities to those described in various inflammatory dermatoses; second, we will discuss chronic and antibody-mediated rejection (AMR) in VCA, highlighting the pitfalls of available studies investigating these emerging topics; finally, we will analyze the applicability of emerging concepts and novel topics in transplantation pathology to the field of VCA.

## Acute Skin Rejection in Hand and Face Transplants

### Review of clinical, cellular, and molecular findings

In 1980, Dvorak et al.’s initial experimental work on rejection of vascularized human skin allograft demonstrated that microvascular endothelium is the critical target of the immune response, and that rejection manifests largely by vascular damage followed by ischemic infarction (6). They provided further evidence that, along these vascular changes, both major T-cell subsets, CD4^+^ (helper/inducer), and CD8^+^ (cytotoxic/suppressor), infiltrate the skin forming perivascular cuffs (7). These eventually penetrate the epidermis and lead to dyskeratosis of epidermal and adnexal keratinocytes (6).

This model is partly similar to a VCA with rejection histological changes appearing initially in form of perivascular infiltrates in the dermis; however, major immunologic differences exist. First, a variety of immunosuppressive and immunomodulatory protocols are used in VCA (8) which impacts the dynamics rejection; second, the skin, being transplanted with other components in a VCA, is rendered less antigenic (9) which might alter the timing and intensity of rejection episodes.

In this regard, a comprehensive understanding of the basic cellular and molecular dynamics of rejection in VCA is crucial for demystifying rejection mechanisms and ultimately devising accurate and specific diagnostic measures.

#### Hand transplantation

In hand transplantation, acute rejection manifests by changes either in the skin or less often in the palm and nail beds.

##### “Typical” picture

###### Macroscopic features

Macroscopic features of skin rejection include a maculopapular erythematous rash of diverse color intensities. It may be diffuse, patchy or focal, and with or without burning pain (10–12). It is distributed over the dorsal and volar aspects of the forearm and wrist, and the dorsum of the hand. This represents the “classical” pattern of rejection, sparing palmar skin and nails.

###### Microscopic features

As for the microscopic features, these are summarized by the Banff classification of hematoxylin-/eosin-stained sections (5): Grade I includes mainly lymphocytic perivascular aggregates in the dermis. In mild rejection stages, the inflammatory infiltrate is found in the interstitium and interphase between dermis and epidermis and/or adnexal structures. Moderate rejection is characterized by cellular infiltrate in the epidermis. Advanced stages are characterized by necrosis of keratinocytes resulting in focal dermal-epidermal separation, and finally necrosis with loss of the epidermis.

###### Immunohistochemical features

Immunohistochemically, the infiltrate in acute skin rejection is comprised predominantly of CD3^+^ T-cells spreading with progression of rejection from the perivascular space to the dermis and epidermis. Among CD3^+^ cells, a tendency toward a predominance of CD8^+^ T-cells in milder cases and CD4^+^ T-cells in advanced cases is observed. Furthermore, depending on the grade of rejection, 10–50% stain positive for CD68, a total of 0.5–5% for CD20 and CD79a, and about 5–10% for FoxP3 (13). As for Indoleamine 2,3-dioxygenase (IDO) positive cells, these are mostly found scattered in the dermis in grade I rejection (14). The involvement of Foxp3 and IDO in promoting tolerance in VCA remains speculative although some studies suggested a synergistic role of both molecules in the setting of CTLA4 Ig induced tolerance in murine cardiac allografts (15).

##### “Atypical” picture

An “atypical” pattern of hand rejection has been described involving palmar skin and nail beds. This occurred in patients exposed to repetitive and persistent mechanical stress of the palm (16).

###### Macroscopic features

Macroscopic features consist of fingers swelling and a desquamative rash associated with dry skin, red papules, scaling, and lichenification of the palm. Burning pain was reported in a minority of patients. Nail involvement is characterized by dystrophy, degeneration, deformation, or even loss. Worth noting that there were some erythematous lesions affecting the forearm and/or the dorsum of the hand (typical picture features). These however resolved spontaneously, or responded to conventional treatment, whereas those on the palm did not (17).

###### Microscopic features

On the microscopic level, biopsies of palmar skin showed variable degree of lymphocytic infiltrate. That commenced in the perivascular and perineural areas of the dermis (grade 1), and progressed into the superficial dermis with erosion of basal epidermis in some cases. There was also epidermal “hyperkeratinization,” with evidence of spongiosis and cytoid bodies. As for the nail bed, biopsy showed a lymphocytic infiltrate, similar to what was observed in the palmar skin (17).

###### Immunohistochemical features

Immunohistochemistry revealed a prevalence of T-cells (CD3^+^), together with a small number of B-cells (CD20^+^ and CD79a). The T-cell infiltrate predominantly comprised CD4^+^ T-cells and, to a lesser extent, CD8^+^ T-cells. There was a minority of CD68^+^ macrophages and FoxP3^+^ cells, and no C4d staining.

#### Face transplantation

In face transplantation, the high antigenicity of the oral/nasal mucosa compounds the immunologic challenge imparted by the skin. In a minority of cases, a sentinel skin graft (SSG) from the donor was transplanted for surveillance biopsies and monitoring of clinicopathologic signs of graft rejection (2, 18).

##### Macroscopic features

These include skin redness, swelling, and appearance of nodules and papules (19, 20). The oral mucosa is erythematous, and a SSG, when present, will display diffuse edema and erythema. In this situation, since the appearance of the facial graft (red macules) is different than that of the SSG (diffuse redness), it is important to differentiate rejection from various facial dermatoses manifesting with erythema (seborrheic dermatitis, rosacea, psoriasis, contact dermatitis, etc…). A Periodic acid-Schiff stain of the oral mucosa is recommended not to miss a fungal infection (21).

##### Microscopic features

Microscopically, pathologic changes seen in skin and mucosal biopsies during rejection are qualitatively similar to those observed in hand rejection. The dermis shows edema and a predominantly lymphocytic inflammatory infiltrate of variable density; in the surface epithelium (epidermis or mucous membrane), intercellular edema, lymphocyte exocytosis, basal cell vacuolization, and keratinocyte apoptosis are noted (13). The severity of these changes can be assessed according to the same scoring system proposed for hand transplantation (22). Interestingly, biopsies of the oral mucosa show more severe changes than those seen on the SSG and the facial graft (23, 24). The explanation for this observation is unclear; it could be due to a higher density of antigen-presenting cells (dendritic and Langerhans cells) in the mucosa as opposed to skin.

##### Immunohistochemical features

Immunohistochemical studies showed that T-cells infiltrating the facial graft, oral mucosa, and SSG expressed predominantly a CD4^+^ phenotype, with fewer cells expressing CD8^+^. Most biopsies contained within the perivascular dermal infiltrate a small percentage (about 10%) of cells expressing cytotoxic associated TIA-1 antigen. FoxP3^+^ T-regulatory cells (T-regs) were found in small amounts (10–15% of the infiltrate) in most mucocutaneous biopsies, mainly within the dermis but occasionally in the surface epithelium whenever significant exocytosis was present. An admixture of occasional CD20^+^ B-cells was found in some biopsies too (13).

### Analysis of clinical findings

Interestingly, from a clinicopathological view, changes seen during facial allograft rejection were similar to those observed during rejection of other skin-containing composite allografts, such as hand (25, 26) and also abdominal wall (27), suggesting that rejection manifests microscopically in the skin in a rather similar way. Similarly, with respect to cellular dynamics, these were also comparable in hand and face transplantation, consisting predominantly of CD3^+^/CD4^+^ T-cells, with a lower percentage of CD8^+^ or TIA-1^+^ cytotoxic T-cells (28).

#### Immune mechanisms of acute skin rejection

Understanding the immune mechanisms of acute skin rejection aids in making an early diagnosis and delivering appropriate treatment. Knowledge of these mechanisms is also critical in developing strategies to treat/reverse rejection and in devising new immunomodulatory therapeutic strategies to ensure longer survival of these transplants. We will provide below a brief overview of this topic as this is not the main focus of our review.

It is well known that the complex machinery of transplant rejection is orchestrated by T-cell adaptive alloimmune responses (29). The typical acute rejection usually occurs a few days after transplantation, the time necessary for the activation, proliferation, and differentiation of T-cells. This immune response is dependent on three signals: alloantigen recognition, activation of T-cells, and signal for T-cell proliferation (30). Alloantigen recognition (the first signal) can occur by three different mechanisms: (a) Direct pathway: alloreactive T-cells directly recognize intact allogeneic MHC molecules expressed on donor cells (namely APCs) (31). (b) Indirect pathway: alloreactive T-cells recognize processed allogeneic MHC molecules expressed on recipient APCs (32). (c) Indirect pathway: alloantigens are transferred from donor APCs to recipient APCs, through cell–cell contact or through transfer of donor exosomes (33). It is presumed that the direct pathway is the major cause underlying rejection occurring early on post-transplantation; its contribution to late rejection is minimal. The indirect pathway is the main player in later rejection. The role of the semi-direct pathway in triggering rejection episodes at specific time points post-transplantation remains to be determined.

Emerging research on mechanisms of transplant rejection is taking a different approach by focusing on the contribution of the innate myeloid cells to the development of transplant immunity. There is now evidence that the innate immune system of the recipient modulates adaptive immune responses through activation of a number of cells, ligands, receptors, transcription factors, chemokines, and cytokines (34). Other factors, namely ischemia-reperfusion injury (35) and complement system activation (36), come into play and modulate the rejection alloimmune reaction.

With regard to the cellular and molecular players inducing skin rejection specifically, a neutrophil-mediated stimulation and progression of acute rejection, described previously in murine cardiac grafts (37), has been suggested as one of the relevant mechanisms in VCA. Also, endothelial IDO and adhesion molecules on the endothelium of graft vasculature (ICAM-1, E-selectin, and LFA-1) were shown to be associated with the presence and severity of rejection. The inhibition of E- and P-selectin by Efomycine M in a rat hindlimb transplantation model, and the subsequent allograft acceptance underlines their role in promoting skin rejection (14).

#### Non-specificity of acute skin rejection features

Pathological changes of skin rejection in VCA evolve according to the severity of rejection; although characteristic, they are non-specific and closely resemble several inflammatory dematoses (21). Effectively, grade I rejection presenting clinically with minor erythema, and pathologically with mild perivascular infiltrates resembles manifestations of mild viral infections. The picture gets further complicated in grade II rejection as the differential diagnosis of grade II features is much broader. The moderate erythematous changes sometimes seen with scaling, and associated with denser perivascular infiltrates (with or without epidermal exocytosis/spongiosis) can also occur in viral infections, dermatophyte infection, drug allergic reactions, contact dermatitis, as well as insect bites. As for grade III consisting of lichenoid plaques or papules (in the form of hyperkeratosis, hypergranulosis, or acanthosis) and associated with even denser dermal infiltrates affecting the epidermal appendages and reaching the lower epidermal layers mimics the clinical picture of cutaneous pseudolymphomas, or even full blown B-cell lymphomas, and various other dermatoses characterized by lichenoid appearance (lichen planus, lichenoid drug eruptions, erythema multiforme, lupus erythematosus). Grade IV rejection, documented so far in the first human hand transplant only, and consisting of necrotizing events with epidermal necrosis, resembles severe drug reactions (toxic epidermal necrolysis). It is worth noting that presence or absence of these manifestations in the recipient’s own skin might be beneficial in guiding the diagnosis. Also a careful review of the past medical history of the recipient and donor is crucial.

## Chronic Rejection in Hand and Face Transplants

Despite a high incidence of acute rejection in VCA as compared to solid organ transplantation, chronic rejection might be a much rarer event. At present, the exact mechanisms of chronic rejection have not been defined. It was noted however that both immunologic and non-immunologic factors are implicated (38); but still, insufficient data are available to describe VCA features of chronic rejection. The Banff 2007 and 2011 classifications did not include any characteristics of chronic rejection (5, 39). Clinicopathologic features suggestive of chronic rejection could include myointimal proliferation of arterioles, loss of adnexa, nail changes, skin and muscular atrophy, and fibrosis of deep tissues (5). Recently, a group from Lyon (40) has thoroughly assessed four bilateral hand-grafted patients (10, 7, 3, and 2 years of follow-up, respectively) and one facial allotransplantation (5 years of follow-up) by histology, magnetic resonance imaging, ultrasonography, and high resolution peripheral quantitative computed tomography scan. There were no lesions suggestive of chronic rejection, namely dermal fibrosis or vascular stenosis. Similar results were described by the Innsbruck group in their 10 year report update (41). The absence of chronic rejection in VCA might be due to the fact that all episodes of acute rejection were diagnosed at an early stage and treated immediately.

Worth noting that an acute arterial thrombosis due to intimal hyperplasia in a unilateral hand-grafted recipient was reported to occur 275 days after transplantation; although the patient had four episodes of untreated acute rejection, the cause of that thrombosis was not well defined (42). These untreated acute rejection episodes might actually be the cause, since in experimental rat hindlimb models, changes such as intimal hyperproliferation and luminal narrowing/occlusion consistent with chronic rejection or allograft vasculopathy were shown to occur after repeated episodes of acute skin rejection and frequent lapses in maintenance immunosuppression (43). Of note, this study, investigating tissue-specific pathological changes secondary to multiple AR episodes in complete mismatch transplants, does not accurately reproduce a chronic rejection model; effectively, the mechanism of rejection is thought to be a continuous subclinical process as opposed to multiple episodes of clinically overt rejection. Similarly, in another study of chronic rejection histopathology in a non-human primate model of VCA, Mundinger et al. were also able to detect transplant vasculopathy features in their model of chronic rejection (44); however, this model, also relying on complete mismatch transplants treated with full immunosuppression followed by complete withdrawal of medications 200 days later, does not accurately reproduce the immune reaction responsible for a chronic rejection phenomenon.

In humans, Kaufman et al. reported some degree of transplant vasculopathy in six of their hand transplant recipients, with aggressive and severe intimal hyperplasia observed early post-transplant in two patients (45). True that vascular lesions such as intimal proliferation and luminal occlusion are reminiscent of chronic rejection in solid organ transplantation, however it is still difficult to affirm that acute ischemia of the grafted upper extremity in the absence of other previous signs, is a clinical manifestation of chronic graft rejection in VCA (42). Clearly, there is an urgent need for appropriate animal models to study chronic rejection in VCA. In solid organ transplantation, Fisher to Lewis rat transplantation is regarded as a well-established and reproducible model for chronic allograft rejection (46). In such models the histopathological changes observed in the rejecting renal allograft were comparable to those observed during chronic rejection in humans (47). Using this model in VCA might provide a solid platform for analyzing the underlying immune and non-immune mechanisms involved in chronic rejection pathogenesis.

## Antibody-Mediated Rejection in Hand and Face Transplants

While cellular mediated rejection in VCA has been largely studied, AMR is not well described. In some hand transplant protocol biopsies, C4d complement deposition was evaluated in an effort to examine AMR. These were found in approximately 50% of all skin biopsies, but no correlation with graft function or cellular rejection could be established (48–50). Additionally, vascular C4d deposits may be found in inflammatory dermatoses unrelated to rejection (51). Hence, the reproducibility, sensitivity, and specificity of C4d as the advocated marker for AMR is limited. This causes a considerable number of AMR cases to be C4d negative, and thus not meeting current Banff criteria (39). Other markers of AMR are worth evaluating, namely IgG, IgM, IgA, C1q, C3d, and especially anti-HLA class I and class II (also termed donor-specific antibodies, DSA). DSA were found in some hand transplant recipients, but did not correlate with C4d deposition in skin biopsies. Recently, a study using a rat heterotopic hindlimb transplant model showed that rejection is accelerated but does not occur hyperacutely in the presence of allosensitization and preformed DSA. This type of rejection was mainly cell mediated, and differed mechanistically from that observed in solid organ transplants (52). Although AMR has been identified as a major cause for allograft failure in renal transplantation (53), the long-term effects of these antibodies in VCA remain to be elucidated. Presently, little evidence points to a significant role of AMR in VCA (54). The lack of knowledge in AMR aspects represents a major obstacle for conducting clinical trials to develop adapted treatment protocols for sensitized patients suffering from debilitating limb injuries and severe disfigurement. This cutting-edge area of transplantation pathology needs suitable animal models for further investigations of rejection mechanisms. Valuable lessons are to be learned from the solid organ transplantation literature (55), and we believe that a rat orthotopic himdlimb partial mismatch transplant using allosensitized recipients might be a viable option for studying AMR in VCA.

## Novel Concepts in Diagnosing Rejection in Hand and Face Transplants

Although the diagnosis of skin rejection in VCA is guided by an internationally standardized system (5), inherent shortcomings related to histophathological reading and interpretations exist. It is widely recognized that no histological lesion observed in a skin biopsy from a VCA is absolutely specific for a given diagnosis. Furthermore, this method of diagnosis is largely subjective, in that different clinicians (or even the same physician on different days) may report different interpretations of the same biopsy (56). Not only skin rejection lesions are non-specific and mimic several inflammatory, infectious, and some neoplastic dermatoses (as discussed above) (21), intra- and interobserver reproducibility, particularly at the interface between borderline rejection and acute rejection exist (57), and these pose a great diagnostic challenge. The lack of a reliable approach for diagnosing and grading skin rejection in VCA represents a significant unmet clinical need. Objective diagnostic methods such as biological markers might be helpful. In this regard, a considerable amount of ongoing research is directed at establishing reliable serologic or cellular markers that could indicate rejection and even correlate with long-term graft outcomes (much as in kidney or liver transplantation). Effectively, over the last decade numerous studies have applied omics technologies to transplant biopsies or body fluids obtained from transplant recipients with the aim of discovering more precise diagnostic, predictive, and even prognostic biomarkers (58, 59). Other directions are engaged in devising novel strategies centered on cross-disciplinary approaches combining medicine, immunology, mathematics, and computer science. These aim at demystifying the biological processes of rejection in an effort to establish predictive algorithms for the different grades of rejection based on cytokines expression levels (60). In these novel approaches, different sample types might be used for analysis. Ideally, it is best to use starting material that is as close to the site of rejection as possible. This may include graft tissue, fluid that might be potentially collected close to the site of rejection, serum/plasma, or even urine. It is worth mentioning that the concentration of rejection biomarkers (sampled from the graft) will decrease as one moves further away; thus it is highest in the graft tissue, lower in interstitial fluid, less in serum/plasma, and might be undetectable in urine. Noteworthy, that this situation might be reversed in cases of advanced rejection where graft cellular apoptosis results in pouring of cells’ contents in the blood increasing their concentration peripherally. Therefore, careful standardization of sampling conditions, i.e., timing but also sampling site (graft [volar vs. palmar, distal vs. proxiam], serum, urine) is of utmost importance for obtaining reproducible results (61). When the ultimate goal is identifying rejection markers that are readily translatable to clinical trials, analyzing blood samples (plasma/serum) is most useful. In fact, serum is typically preserved in all clinical laboratories, and all the infrastructure to separate it and analyze it is present in most academic institutions.

However, all these new diagnostic methods might face great challenges on their way to clinical use. In fact, since there is no diagnostic gold standard against which the obtained results can be validated, any candidate set of biological molecules (whether DNA, RNA, cytokines, or other proteins) will require independent validation in larger prospective studies with different samples (but using the same platform for initial discovery). Therefore, the integration of new diagnostic tests and platforms into existing consensus classifications will need close collaboration between clinicians, pathologists, molecular biologists, biostatisticians, and omics specialists. It is through this interdisciplinary collaboration that diagnostic precision will be continuously increased in transplantation pathology.

## Conclusion

Vascularized Composite Allotransplantation has emerged over the past decade as a novel reconstructive and restorative option for patients with severe tissue defects and disfigurement. Despite initial incertitude, outcomes exceeded all expectations at the immunologic, functional, and cosmetic levels. Rejection remains however a serious threat. Innovative strategies aiming at a better understanding of the dynamics and immunology of rejection in VCA are needed. The real advances will come from studying the molecular and cellular mechanisms that underlie and precede rejection, the impact of these mechanisms on long-term graft outcomes, and the other molecular and cellular changes that impede the development of donor-specific tolerance. Findings from these studies are likely to have a broader impact on organ transplantation in general. We have already seen a flow of interest in multi-disciplinary approaches for devising accurate and reproducible diagnostic strategies for rejection in VCA. Although the ultimate goal of these studies is to improve the diagnosis of rejection and even build predictive models, it will be equally important to develop appropriate animal models in which to test different aspects of rejection at various times post-transplantation. These animal models need to recapitulate similar changes and challenges seen in VCA rejection. To this end, no objective measure of rejection exist; diagnosis remains largely dependent on clinicopathologic cues which are overall non-specific. Novel concepts and technologies for improving this aspect of VCA are ongoing and hold great promise in advancing the development and widespread application of a safer VCA, favoring the risk-benefit for such life-changing transplants.

## Conflict of Interest Statement

The authors declare that the research was conducted in the absence of any commercial or financial relationships that could be construed as a potential conflict of interest.
